# Glandular odontogenic cyst mimicking central mucoepidermoid carcinoma

**DOI:** 10.4103/0973-029X.64303

**Published:** 2010

**Authors:** Sudeendra Prabhu, K Rekha, GS Kumar

**Affiliations:** *Department of Oral and Maxillofacial Pathology, SDM College of Dental Sciences, Dharwad, Karnataka, India*; 1 *Department of Oral Pathology, K.S.R. Institute of Dental Science and Research, Tiruchengode, Tamil Nadu, India*

**Keywords:** Glandular odontogenic cyst, mucoepidermoid cyst, sialoodontogenic cyst

## Abstract

Glandular odontogenic cyst (GOC) is a rare developmental cyst of the jaws. The most common site of occurrence is the anterior mandible, and it is most commonly seen in middle-aged people. It is a destructive lesion with a high rate of recurrence. A predilection for men is observed. Clinical and radiographic findings are not specific, and it can mimic as any other destructive lesion of the jaw. The histopathological features of GOC and those of low-grade central mucoepidermoid carcinoma (MEC) are similar. Often, they are misdiagnosed as MEC. We present a case of GOC in the posterior maxilla, which is a rare site. The similarities and differences between GOC and central MEC are also discussed.

## INTRODUCTION

The glandular odontogenic cyst (GOC) is a rare developmental cyst of the jaws that was described in 1988 by Gardner *et al*.[1] as a distinct entity. The first two patients with features of GOC were reported by Padayachee and Van-wyk in 1987.[2] It is also known as sialoodontogenic cyst, mucoepidermoid cyst (MEC)[3] or polymorphous odontogenic cyst.[4] Its name was changed to GOC by Gardner *et al*. because of the lack of evidence of salivary gland origin, and the term was later adopted by the World Health Organization (WHO).[5]

GOC is rare, with just over 71 cases reported in the literature.[5] The most common site is the anterior mandible, (88%) and it mostly occurs in middle-aged people (40–60 years) with a male predilection. Recurrences have been described in 30% of the cases.[6,7]

Clinical findings are not specific and an asymptomatic swelling is frequently observed. A unilocular or multilocular well-defined radiolucency is usually seen.[8] There is similarity and overlap between the microscopic features of the GOC and those of low-grade, predominantly cystic, central mucoepidermoid carcinoma (MEC).[9]

The present case shows all the characteristic features of a GOC but, histopathologically, mimics central MEC.

## CASE REPORT

A 47-year-old female patient was seen, complaining of swelling in the right upper back region of the jaw since 2 months. She underwent extraction of a tooth in the same region 7 months back. Intraoral examination revealed a swelling extending from the right premolar region to the tubersity. Expansion of both cortical plates was evident. Sinus opening was present in relation to the extraction socket of the maxillary right first molar. The radiograph showed a well-defined unilocular radiolucency in the right posterior maxilla. The lesion was enucleated along with involvement of extraction of the tooth. The surgical specimen consisted of the soft tissue, which was cystic, grayish in color, with irregular borders, rough surface and soft in consistency.

**Figure 1:**
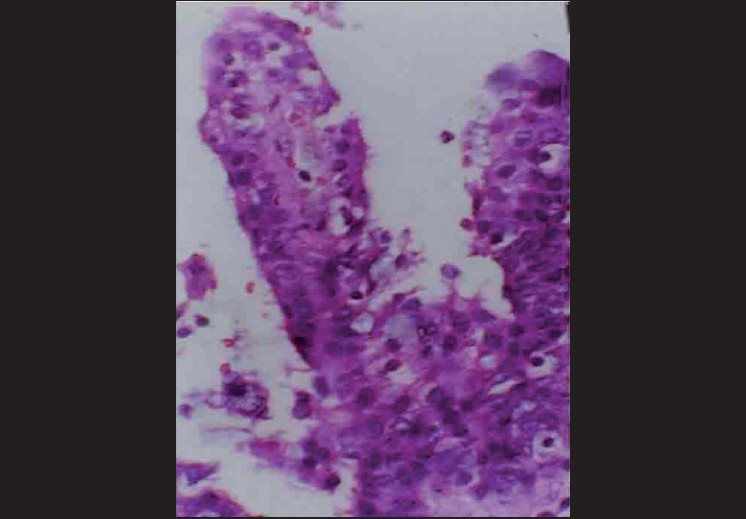
Papillary luminal proliferation with intraepithelial crypts filled with mucin

**Figure 2:**
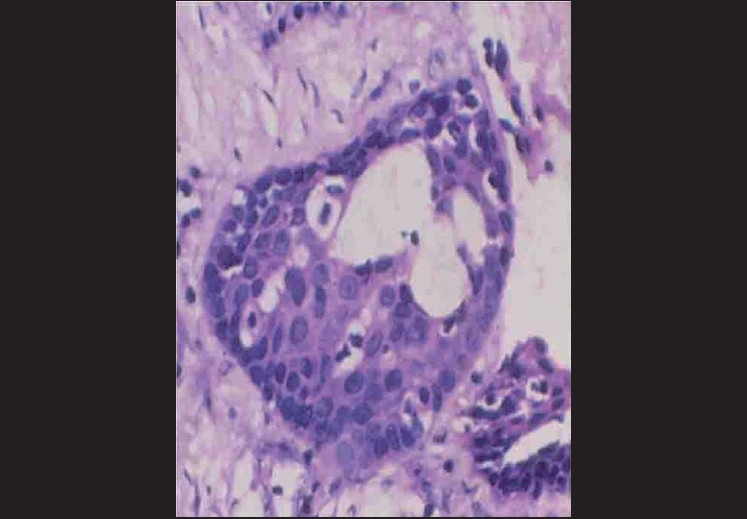
Epithelial plaque showing clear cells and epidermoid-like cells

**Figure 3:**
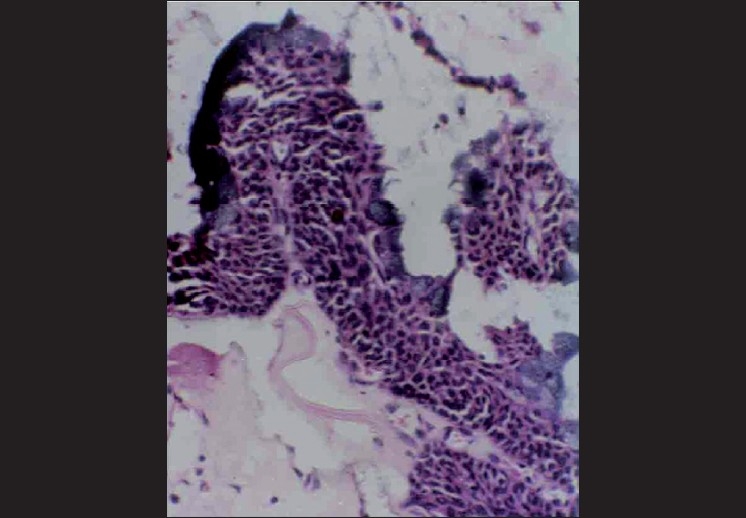
Epithelial sphere or epithelial plaque showing mucous cells

**Figure 4:**
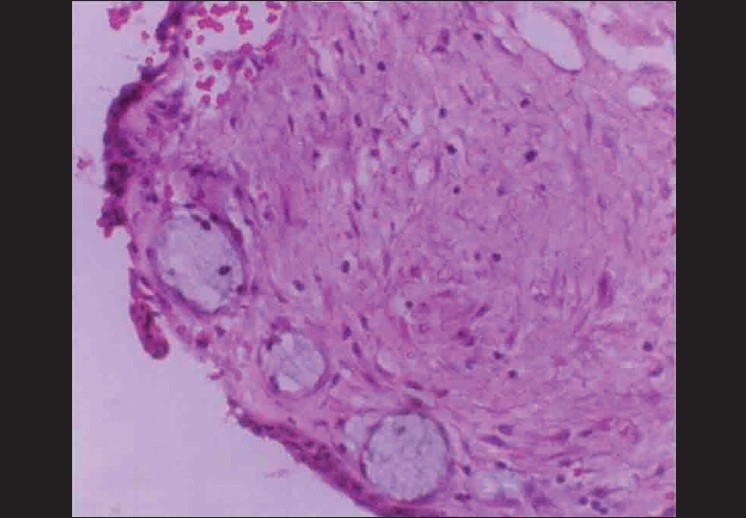
Cystic spaces in capsule filled with mucin

Microscopically, the specimen showed a nonkeratinized epithelial lining with areas of papillary growth enclosed with intraepithelial crypts that were either empty or filled with mucin [Figure 1]. The cystic lining was thin and consisted of cuboidal cells resembling reduced enamel epithelium. In certain areas, the cyst lining was continuous with the sinus lining. Many mucous cells, clear cells and few cells resembling that of epidermoid cells were also observed [Figure 2]. Focal areas depict epithelial thickening or plaque formation [Figure 3]. Many cystic spaces filled with mucin were evident in the fibrous connective tissue capsule [Figure 4].

**Figure 5:**
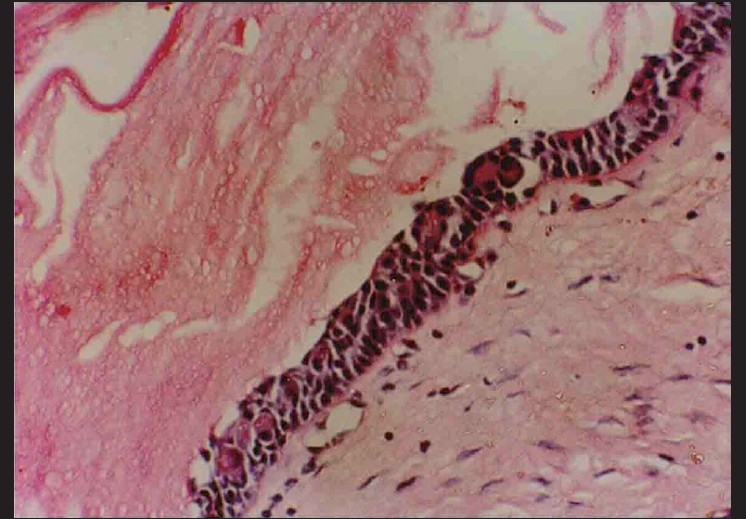
Mucicarmine stain: Mucous cells and mucous pooling showing positivity

**Figure 6:**
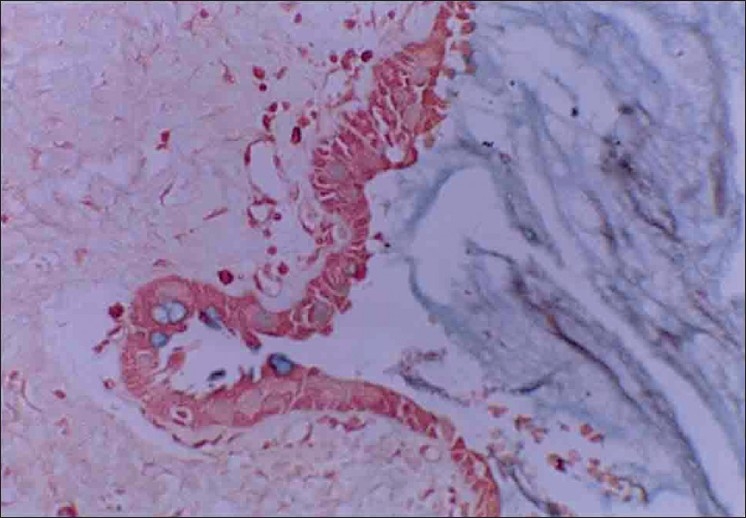
Alcian blue stain (acetate buffer, pH 2.5) showing positivity for mucous cells and mucous pooling

**Figure 7:**
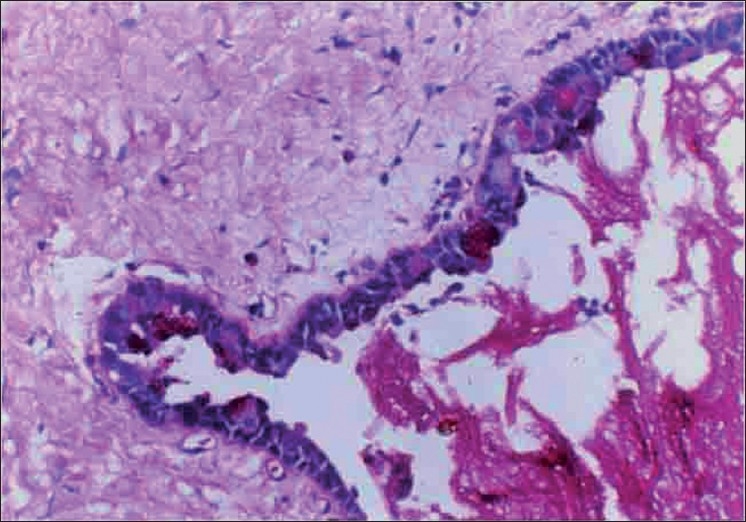
PAS stain: Clear cells showing positivity (without diastase)

**Figure 8:**
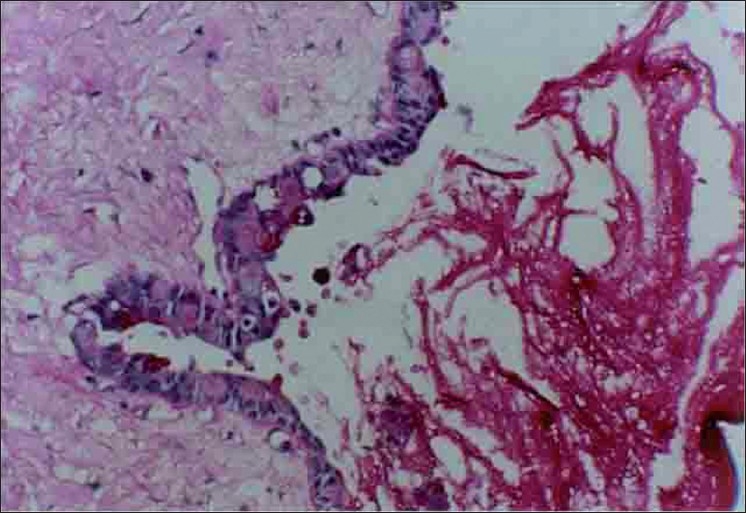
PAS stain: Clear cells becoming empty again after using diastase (indicating the presence of glycogen)

The presence of mucin was confirmed by mucicarmine staining [Figure 5]. Mucin was characterized using PAS and alcian blue. Mucous cells and mucous pooling showed positivity to alcian blue and PAS, suggestive of acidic mucin [Figures 6 and 7]. Clear cells were positive for PAS without diastase and negative for PAS with diastase, indicating their content of glycogen [Figures 7 and 8]. The final diagnosis of glandular odontogenic cyst was given. The patient is now being followed-up regularly and, in the past 5 years, there has been no evidence of recurrence.

## DISCUSSION

GOC is a rare cyst of the jaws. It appears to be a distinct entity because of its characteristic histopathologic features. The anterior mandible is the most common site of occurrence of this cyst.[1] Incidence in the maxilla is rare. However, cases that were reported were from the anterior region.[7] But, in this case, the lesion was found in the posterior maxilla.

The present case has all the characteristic features of GOC given by Gardner *et al*.,[1] such as presence of thin cystic lining with cuboidal cells and papillary luminal proliferation. Intraepithelial crypts with mucicarmine-positive material and presence of characteristic epithelial plaque was also evident.

According to Magnusson *et al*.,[8] the central MEC, especially the low-grade variant is regarded as the most important histopathological differential diagnosis from GOC. Features in our case common with low-grade central MEC are the presence of epidermoid, mucous and clear cells along with cystic spaces filled with mucin.

It has been speculated that GOC may represent the most benign end of the spectrum of central MEC.[8] But, according to Waldron and Koh,[10] the distinguishing feature in GOC is the typical thin epithelial lining without any solid epithelial proliferation as seen in MEC. In addition, MEC do not show the swirling spherical aggregates (epithelial plaque) that are often seen in GOC.[9] The immunohistochemical examination performed by Semba *et al*.[11] for expression of cytokeratins and epithelial membrane antigen suggested that the lining of the epithelium was of odontogenic origin with metaplastic mucous-laden cells.

Our case is finally considered as GOC as it fulfills all the criterias given by Gardner *et al*.[1] and, unlike MEC, cellular atypia, solid and microcystic epithelial proliferation were not seen.

The treatment of choice is controversial because of the few reported cases and method suggested varies from curettage to local block excision. A long-term follow-up of 5 years is advised as it has a high rate of recurrence. Prognosis is good. In the present case, enucleation was performed and there was no recurrence since the last 5 years.
